# Effects of Combinatorial Treatment with Pituitary Adenylate Cyclase Activating Peptide and Human Mesenchymal Stem Cells on Spinal Cord Tissue Repair

**DOI:** 10.1371/journal.pone.0015299

**Published:** 2010-12-20

**Authors:** Kuan-Min Fang, Jen-Kun Chen, Shih-Chieh Hung, Mei-Chun Chen, Yi-Ting Wu, Tsung-Jung Wu, Hsin-I Lin, Chia-Hua Chen, Henrich Cheng, Chung-Shi Yang, Shun-Fen Tzeng

**Affiliations:** 1 Department of Life Sciences, National Cheng Kung University, Tainan, Taiwan; 2 Center for Nanomedicine Research, National Health Research Institutes, Zhunan, Taiwan; 3 Medical Research and Education, Taipei Veterans General Hospital, Taipei, Taiwan; 4 Department of Surgery, School of Medicine, National Yang-Ming University, Taipei, Taiwan; 5 Neural Regeneration Center, Taipei Veterans General Hospital; 6 Department of Pharmacology, School of Medicine, National Yang-Ming University, Taipei, Taiwan; 7 Graduate Institute of Biomedicine and Biomedical Technology, National Chi-Nan University, Puli, Taiwan; University of Sao Paulo, Brazil

## Abstract

The aim of this study is to understand if human mesenchymal stem cells (hMSCs) and neuropeptide pituitary adenylate cyclase-activating polypeptide (PACAP) have synergistic protective effect that promotes functional recovery in rats with severe spinal cord injury (SCI). To evaluate the effect of delayed combinatorial therapy of PACAP and hMSCs on spinal cord tissue repair, we used the immortalized hMSCs that retain their potential of neuronal differentiation under the stimulation of neurogenic factors and possess the properties for the production of several growth factors beneficial for neural cell survival. The results indicated that delayed treatment with PACAP and hMSCs at day 7 post SCI increased the remaining neuronal fibers in the injured spinal cord, leading to better locomotor functional recovery in SCI rats when compared to treatment only with PACAP or hMSCs. Western blotting also showed that the levels of antioxidant enzymes, Mn-superoxide dismutase (MnSOD) and peroxiredoxin-1/6 (Prx-1 and Prx-6), were increased at the lesion center 1 week after the delayed treatment with the combinatorial therapy when compared to that observed in the vehicle-treated control. Furthermore, in vitro studies showed that co-culture with hMSCs in the presence of PACAP not only increased a subpopulation of microglia expressing galectin-3, but also enhanced the ability of astrocytes to uptake extracellular glutamate. In summary, our in vivo and in vitro studies reveal that delayed transplantation of hMSCs combined with PACAP provides trophic molecules to promote neuronal cell survival, which also foster beneficial microenvironment for endogenous glia to increase their neuroprotective effect on the repair of injured spinal cord tissue.

## Introduction

A traumatic primary injury to the spinal cord (SCI) induces axonal degeneration, neural cell death, and microvasculature destruction. These events subsequently trigger a cascade of pathological actions (so called secondary damage) including vascular and biochemical changes, hemorrhagic necrosis, inflammatory process and demyelination [1], [2], leading to a second wave of cell death and lesion area extension which impair the affected body functions. Moreover, poor trophic support environment of the adult central nervous system (CNS) is hostile to endogenous spinal cord regeneration. The findings from recent biomedical research have indicated promising cell therapies for SCI treatment by utilizing various types of multipotent stem cells such as embryonic stem cells, neural stem cells, mesenchymal stem cells/bone marrow stromal cells (MSCs/BMSCs), adipose tissue-derived mesenchymal stem cells, and umbilical cord blood cells [3], [4], [5], [6].

Human MSCs/BMSCs are multipotent stem cells which can differentiate into several tissue cell types such as neural cells, adipocytes, chondrocytes, osteoblasts and hematopoiesis-supporting stroma, thereby making hMSCs/hBMSCs as promising candidates for regenerative medicine. Moreover, hMSCs/hBMSCs are beneficial for the purpose of autologous transplantation, raising the promising possibility that the cells can be used for stem cell-based approach to treat several neurodegenerative diseases, such as stroke, Parkinson disease, amyotrophic lateral Sclerosis, Alzheimer disease, and SCI [7]. Cumulative evidence shows that the transplantation with BMSCs into injured spinal cord caused axonal growth in the lesion site and produced partially functional recovery in SCI rats [5], [8], [9], [10]. The findings from several laboratories have also indicated that BMSCs may play a guiding role in fostering host axons to grow in the grafted spinal cord after being transplanted into the injured spinal cord [11], [12], [13]. Moreover, it has been indicated that delivery of BMSCs 1 week after injury significantly cell survival and improves the hindlimb locomotor function in animals with moderate SCI [12]. These findings point to the promise of bone marrow derived cell-based strategy for potential SCI repair.

Pituitary adenylate cyclase-activating polypeptide (PACAP), a member of the vasoactive intestinal peptide (VIP)/glucagon peptide family, provokes cAMP production and regulates neurogenesis, neuroprotection and axonal regeneration [14], [15], [16], [17]. Our previous studies demonstrated that PACAP increased neural cell survival in the contused spinal cord tissue [18] and induced hMSCs to differentiate into neuron-like cells [19]. This molecule also shows immunomodulatory action on immune cells, such as microglia and macrophages. For example, PACAP can suppress lipopolysaccharide-induced neurotoxicity in mixed neuron/glia culture [20], and it has an inhibitory effect on tumor necrosis factor-alpha (TNFα) production in injured spinal cords [21]. A recent study also indicates that endogenous PACAP mediates regulatory T cell production in the inflamed CNS, which in turn exerts neuroprotection in experimental autoimmune encephalomyelitis [22].

The aim of the study is to evaluate the potential of combinatorial therapy using hMSCs and PACAP for spinal cord tissue repair. The life span of primary hMSCs used in our previous study is short due to replicative senescence [19], [23]. The primary MSCs that are harvested from patients with disease- or age-differences may produce inconsistent results. Accordingly, we used an immortalized hMSC cell line that was generated by transducing with HPV16 E6/E7 genes [23] and further nucleoporated with a plasmid containing human telomerase. The immortalized hMSCs not only were transdiffereniated into neuronal-like cells in response to neurogenic agents, but also highly secreted growth factors, neurotrophic factors, and cytokines/chemokines. Delayed combinatorial treatment with hMSCs and PACAP was observed to significantly improve hindlimb locomotion in SCI rats. Through in vitro co-culture system using the hMSC cell line and primary rat astrocytes, we provided evidence that the hMSC-derived factors increased the production of membrane glutamate transporter-1 (GLT-1) in astrocytes under the influence of PACAP and promoted astrocytic glutamate uptake activity. These results showed that delayed transplantation of hMSCs in combination with PACAP administration synergistically improves microenvironment beneficial for the spinal tissue repair after traumatic SCI.

## Results

### Neurodifferentiation and trophic factor secretion of immortalized hMSCs

**Figure 1:**
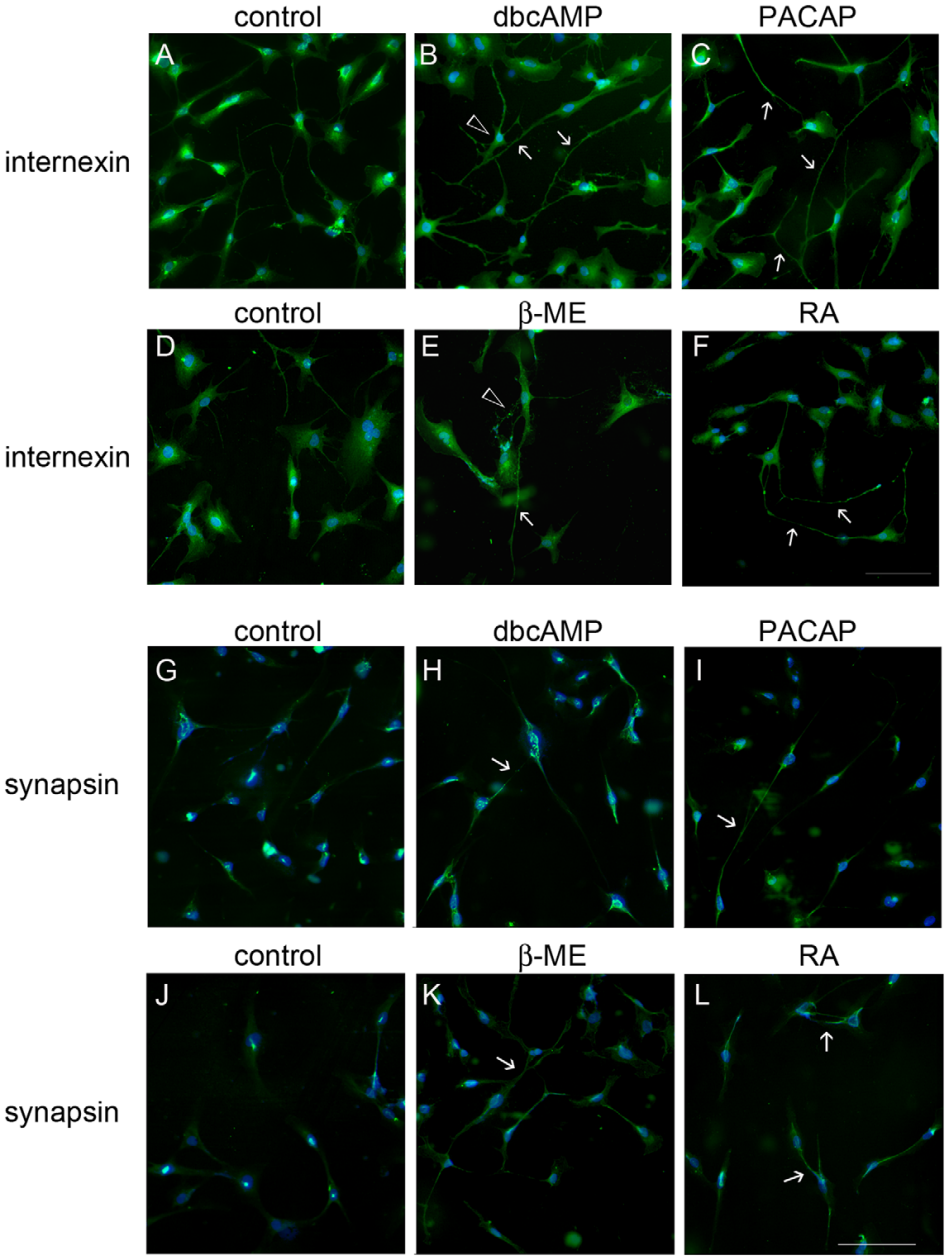
Neuronal-like transformation of immortalized hMSCs after treatment with neurogenic factors. The immortalized hMSCs were treated for 24 hours in serum-free ITS medium with several neurogenic factors, dbcAMP (0.1 mM), PACAP (20 ng/ml), β-mercaptoethanol (β-ME; 1 mM), and retinoic acid (RA; 0.1 µM). The cultures were subjected to immunofluorescence staining for α-internexin (A–F) or synapsin (G–L). Arrows and arrowheads indicate elongation and branching of the processes in hMSCs, respectively. Scale bar in A–L, 100 µm.

The potential of immortalized hMSCs toward neuronal differentiation was first tested. When compared to the control culture, the differentiated cells with positive immunostaining for internexin (a marker for neuronal precursor) and synapsin-1 (a synapse vesicle protein) displayed more elongated processes after treatment with PACAP (20 ng/ml) and other neurogenic agents including dbcAMP, β-mercaptoethanol (β-ME), and retinoic acid (RA) for 24 hours (Figure 1, arrows). In addition, dbcAMP and β-ME induced extensive branching in differentiated hMSCs (Figure 1B and E, arrowheads). The results support the view that the immortalized hMSCs retain the property of their transdifferentiation into neuronal-like cells.

**Figure 2:**
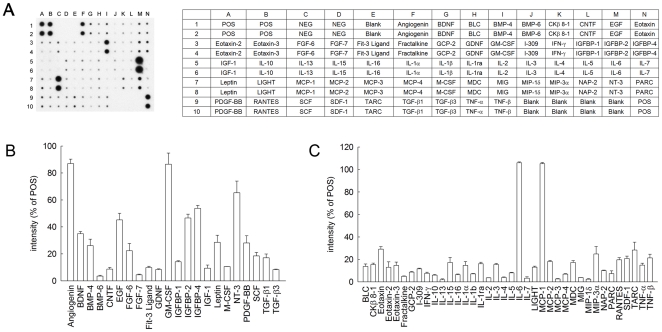
Secretion profile of growth factors and cytokines from immortalized hMSCs. Immortalized hMSCs were cultured in DMEM/LG medium for 48 hours. The cultured media were collected and applied onto a human growth factors/cytokines antibody array membrane (left panel in A). The map of the array that is designed to detect 60 cytokines, chemokines, or growth factors is shown in A (right panel). The arrays were scanned, and the staining intensity of each spot was quantified by densitometry. The percentage of the intensity of the respective spots over that of the positive control (POS) on the same array was represented as the relative expression levels of each human growth factor (B) or cytokine (C). The data represent mean ± SEM of three independent experiments.

Furthermore, through an antibody array analysis (Figure 2), we found that hMSCs used in this study secreted profusely several growth factors, neurotrophic factors, and cytokines, such as angiogenin, epidermal growth factor (EGF), granulocyte/macrophage colony stimulating factor (GM-CSF), insulin-like growth factor-binding protein (IGFBP)-2/4, brain-derived neurotrophic factor (BDNF), bone morphogenetic proteins-4 (BMP-4), neurotrophin-3 (NT-3), platelet derived growth factor-BB (PDGF-BB), interleukin-6 (IL-6), and monocyte chemoattractant protein-1 (MCP-1). The results indicate that hMSCs used in this study can serve as a trophic factor-producing source that may assist tissue repair in the injured spinal cord.

### Basso Beattie Bresnahan score increased by combinatorial treatment with PACAP and hMSCs

**Figure 3:**
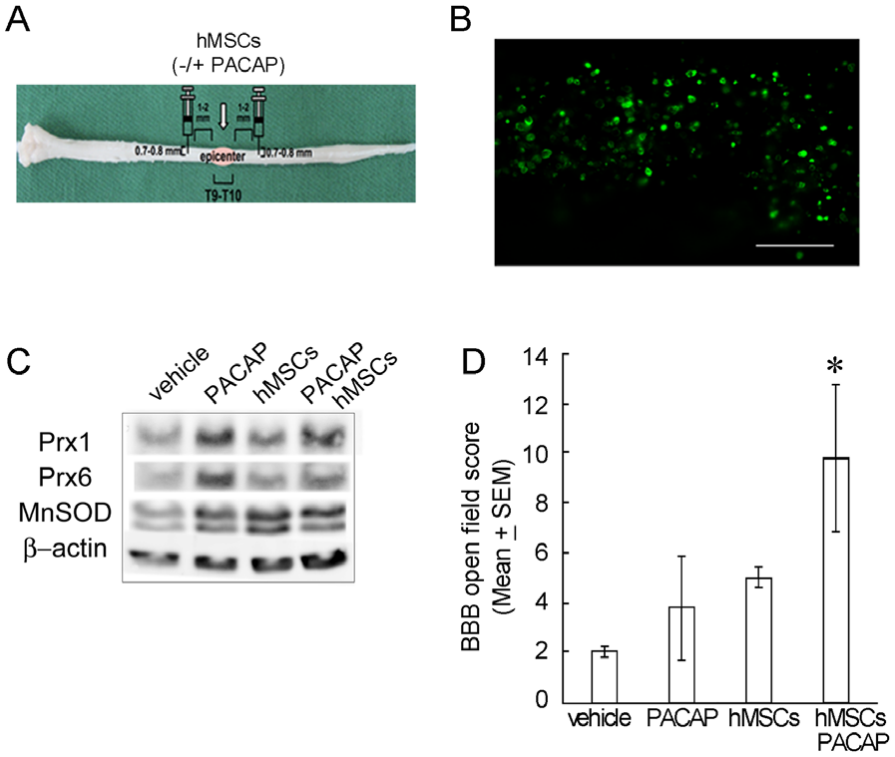
Antioxidant proteins and hindlimb locomotion increased by delayed combinatorial therapy of PACAP and hMSCs. (A). The diagram shows the location of the administration of hMSCs and PACAP into 1–2 mm rostral and caudal to the lesion center at day 7 post severe SCI. (B) Immunostaining for human nucleus (HuNu) was carried out to identify transplanted hMSCs (green) at day 7 after hMSC transplantation. Many hMSCs were present in the injected zone proximal to the lesion center of the spinal cord. Scale bar in B, 50 µm. (C). The injured spinal cords were collected at day 14 after SCI. The lesion center with the length of 4–5 mm was dissected from the injured spinal cord tissues, subjected to protein extraction. The expression levels of selected antioxidant proteins as indicated above were examined by western blotting. The same blot was stripped and reprobed with β-actin antibodies as internal loading control. (D). At day 31 after SCI, rats with severe SCI were subjected onto an open-field to evaluate their hindlimb locomotion using Basso Beattie Bresnahan (BBB) locomotor rating analysis. Data are presented as mean ± SEM. *p<0.05 versus vehicle, PACPA, or hMSCs.

SCI can induce extreme inflammation in the first week after SCI [1]. The study indicated that administration of rat MSCs one week after SCI led to better survival of transplanted cells than immediate transplantation [12]. Accordingly, delayed transplantation of hMSCs combined with PACAP was conducted at day 7 post SCI (Figure 3A). Immunofluorescence for human nuclei (HuNu) showed that many transplanted hMSCs were still present in the injected site at week 1 post transplantation (Figure 3B), while the transplanted cells were scarcely observed at week 2 post transplantation (unpublished observations of Lin and Tzeng).

Although the in vitro study has shown that hMSCs can undergo neuronal differentiation under the influence of PACAP (Figure 1), we did not detect new neurons derived from hMSCs at the injected site and lesion center at week 1 post transplantation (unpublished observations of Lin and Tzeng). This raises the possibility that the transplanted hMSCs combined with PACAP might rather reconstitute niche in the support of the tissue repair, than contribute to the generation of new neurons in the injured spinal cord. Thus, the profile of differential protein expression in the lesion center was studied using the proteomic analysis. In comparison to the expression levels of proteins involving antioxidation/oxidoreduction in vehicle-treated groups, the intensity of Prx-1, Prx-6, and MnSOD was upregulated by 50% in animals with delayed transplantation of hMSCs combined with PACAP (Table S1 and Figure S1). Indeed, western blotting showed that the levels of the three proteins were increased by delayed treatment by PACAP and hMSCs compared to that in the vehicle-treated group (Figure 3C). Moreover, increased levels of Prx-1 and Prx-6 were observed in PACAP-treated animals compared to that in the group receiving hMSC transplantation or treatment with PACAP and hMSCs (Figure 3C). The results provide evidence showing the effect of the combinatorial treatment on oxidant defense in the injured spinal cord.

The hindlimb locomotor function was assessed by Basso Beattie Bresnahan (BBB) locomotor rating scale at day 31 post SCI. As shown in Figure 3C, the BBB score for the SCI rats receiving delayed transplantation of hMSCs was 5.0±0.4 (n = 4). The values were not biostatically different from those observed in SCI rats with PBS injection. The scores in vehicle-treated rats (2.0±0.1; n = 3) were similar to the ones indicated by the previous studies [24], [25]. Delayed administration of PACAP into the injured spinal cord did not induce significant increase in hindlimb locomotor function when compared to vehicle-treated rats having severe locomotor deficit (Figure 3C). However, the locomotion in SCI rats receiving the delayed combinatorial treatment by PACAP and hMSCs (3.8±2.1) was improved at day 31 post SCI with significantly increased BBB score (9.8±1.2; n = 6), which corresponds to weight-supported plantar steps, but no coordination of forelimb and hindlimb [26].

**Figure 4:**
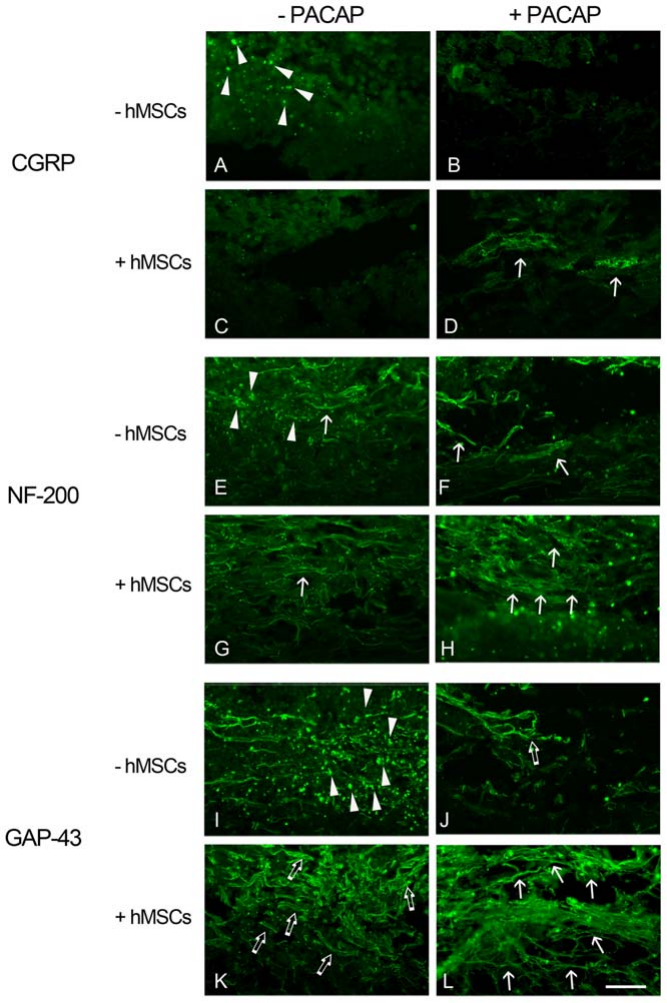
Immunofluorescence staining for identifying the remaining neuronal fibers in the lesion center of the injured spinal cord. After BBB open field scores were performed at day 31, spinal cord tissues were collected. Longitudinal tissue sections were subjected to immunofluorescence for CGRP (A–D), NF-200 (E–H), or GAP-43 (I–L). CGRP positive neuronal fibers were observed in the dorsal section of the spinal cord (D, arrows). Elongated neuronal bundles labeled with CGRP, NF-200, or GAP-43 (arrows in D, H, and L) were observed in the injured spinal cord tissue receiving delayed combinatorial treatment of PACAP and hMSCs. Few NF positive or GAP-43 positive neuronal fibers were seen in the injured spinal cord tissues treated with PACAP (arrows in F and open arrows in G). Fine NF positive fibers (arrow in G) or fragmented GAP-43 positive fibers (open arrows in K) can be detected in the lesion center of the spinal cord with delayed treatment by hMSCs. Arrowheads indicate the cell debris with immunostaining for CGRP (A), NF-200 (E), or GAP-43 (I). Scale bar, 100 µm.

The spinal cord tissue sections collected at day 31 post SCI from the four animal groups were subjected to immunofluorescence to examine the remaining neuronal fibers in the injured spinal cord. Numerous calcitonin gene related peptide (CGRP)-positive neuronal fibers were only found in the dorsal portion of the lesion center of SCI rats receiving the delayed combinatorial treatment by PACAP and hMSCs (Figure 4D, arrows). Note that CGRP-positive neuronal fibers were scarcely observed in the lesion center of vehicle-treated control (Figure 4A), PACAP-treated (Figure 4B) and hMSC-treated animal groups (Figure 4C). Moreover, we found that more orientated elongated neurofilament-200 kDa (NF)-positive neuronal fibers scattered through the lesion center in the injured spinal cord receiving the delayed combinatorial treatment by PACAP and hMSCs (Figure 4H, arrows), when compared to the vehicle-treated control, which had NF-positive neuronal debris (Figure 4E, arrowheads), and collapsed fragmented NF-positive neuronal fibers (Figure 4E, arrow). However, numerous fine fragmented neuronal fibers with NF-positive immunostaining remained in the injured spinal cord tissues treated with delayed infusion by hMSCs (Figure 4F, arrows) or PACAP (Figure 4G, arrows). Furthermore, there was evidence of numerous extending growth associated protein-43 (GAP-43) immunoreactive neuronal fibers in the injured spinal cords proximal to the lesion center in the rats receiving the delayed combinatorial treatment by PACAP and hMSCs (Figure 4L, arrows). However, neuronal fiber fragments with GAP-43 immunoreactivity were observed in the injured center of PACAP-treated or hMSC-transplanted spinal cord (Figure 4J and 4K, open arrows). Note that there was GAP-43-positive cell debris in the injured spinal cord of the PBS-treated control, although a few GAP-43-positive neuronal fibers were also observed (Figure 4I, arrowheads). Together with the behavioral analysis, the high density of the neuronal fibers in the injured spinal cord correlates to partially restoration in the weight support in rats which received the delayed combined treatment by PACAP and hMSCs.

**Figure 5:**
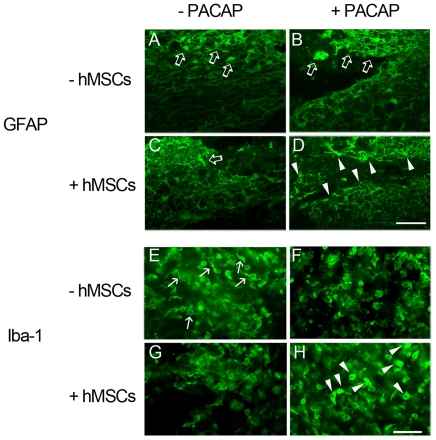
Immunofluorescence of GFAP and Iba-I in the injured spinal cord tissues. The injured spinal cord tissue sections were collected at day 31 after SCI. The longitudinal tissue sections were subjected to immunofluorescence for GFAP (A–D) and Iba-1 (E–H). GFAP positive hypertrophic cells (open arrows) were observed in the areas proximal to the lesion center of the injured spinal cord treated with vehicle (A), PACAP (B), or hMSCs (C), while GFAP positive stellated cells (arrowheads) were found at the periphery to the lesion center treated with hMSCs and PACAP (D). Amoeboid-shaped Iba-1 positive microglia/macrophages (arrowheads) were observed in the injured spinal cord proximal to the lesion center with combinatorial treatment by hMSCs and PACAP (H). Unipolar Iba-1 positive microglia (arrows in E) are present around the lesion center of the spinal cord treated with vehicle. Scale bars, 100 µm (A–D) and 50 µm (E–H).

By glial fibrillary acidic protein (GFAP) immunostaining, we further observed that astrocytes displayed a hypertropic morphology in the injured spinal cord at week 2 post a contusive injury (Figure 5A, arrows). Moreover, the appearance of GFAP-positive astrocytes in the injured spinal cord receiving PACAP (Figure 5B, arrows) or hMSCs (Figure 5C, arrow) was hypertrophic. However, the extension of astrocytic processes was observed in the injured spinal cord with delayed combinatorial treatment by PACAP and hMSCs (Figure 5D, arrowheads). In addition, ionized calcium-binding adaptor molecule 1 (Iba-1) positive microglia in the injured spinal cord by delayed treatment with PACAP and hMSCs displayed an amoeboid-like shape (Fig. 5H, arrowheads), while there were small round Iba-1 stained microglia scattered through the lesion center of the spinal cord in either PACAP-treated (Figure 5F) or hMSC-treated spinal cord (Figure 5G). Note that Iba-1 positive microglia in the vehicle-treated spinal cord tissues mainly displayed a unipolar shape (Figure 5E, arrows). The observations reveal that delayed combinatorial treatment by PACAP and hMSCs exerts effects on the regulation of endogenous glial activities, which may assist tissue repair in the injured spinal cord.

### An increase in galectin-3 expressing microglia by hMSCs and PACAP

**Figure 6:**
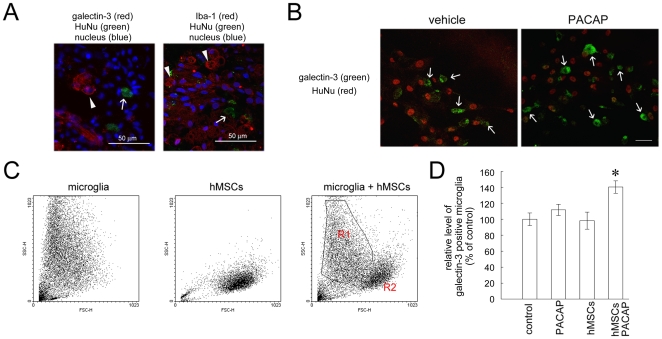
Galectin-3 positive microglia increased by combinatorial treatment with PACAP and hMSCs. (A). The injured spinal cord was collected at day 14 post SCI, which was sectioned and subjected to immunofluorescence staining for galectin-3 (red, arrowhead)/HuNu(green, arrow) and Iba-1 (red, arrowhead)/HuNu (green, arrow). The tissue sections were then incubated with DAPI for nuclear counterstain. (B). Microglia-hMSC co-cultures were treated with PACAP (100 nM) for 24 hours, and then subjected to immunofluorescence staining for galectin-3 (green) and HuNu (red). (C). A representative cytogram of microglia and hMSCs was shown in the left and middle panel, respectively. Accordingly, the cytogram of microglia-hMSC co-culture indicates two cell populations (right panel): microglia scattering in R1, and hMSCs appearing in R2. (D) Microglia were treated with PACAP (100 nM), or co-cultured with hMSCs in the absence or presence of PACAP (100 nM). 24 hours later, the relative levels of galectin-3 positive microglia were determined by FACS. The data represent as the relative level of galectin-3 positive microglia by determining the ratio of galectin-3 positive microglia being analyzed in the region of R1 (C) in comparison with that detected in the control culture. The images shown in B and C were taken from confocal microscopy. Data consist of means ± SEM of three independent experiments. Scale bar in A, 50 µm; in B, 40 µm. *p<0.05 versus control.

Galectin-3/MAC-2, a member of a class of carbohydrate-binding proteins, has been known to play a role in tissue repair following cerebral ischemia [27], [28] and mediates microglial phagocytosis [29], [30]. The proteomic analysis showed an increase in the levels of galectin-3 in the injured spinal cord after the delayed treatment by hMSCs combined with PACAP (Table S1). The recent findings have shown the upregulation of galectin-3 expression in microglia located in the hippocampus receiving hMSCs after transient global ischemia [27]. In our study, galectin-3-positive cells with amoeboid-like cells were observed at the lesion center of the spinal cord receiving delayed transplantation with hMSCs (Figure 6A). Neither galectin-3 nor Iba-1 immunostaining was colocalized to the transplanted hMSCs with HuNu immunoreactivity (Figure 6A, arrowheads). In vitro study using microglia was performed to examine whether PACAP and hMSCs played the regulatory role in microglial galectin-3 expression. As shown in Figure 6B, galectin-3-positive cells without HuNu immunoreactivity were increased in the culture treated with PACAP for 24 hours. The findings were further verified by flow cytometric assay showing that galectin-3 expressing microglia were significantly increased by co-cultured with hMSCs in the presence of PACAP (Figure 6C and 6D).

### Glutamate transporter-1 expression in astrocytes treated with PACAP and hMSCs

**Figure 7:**
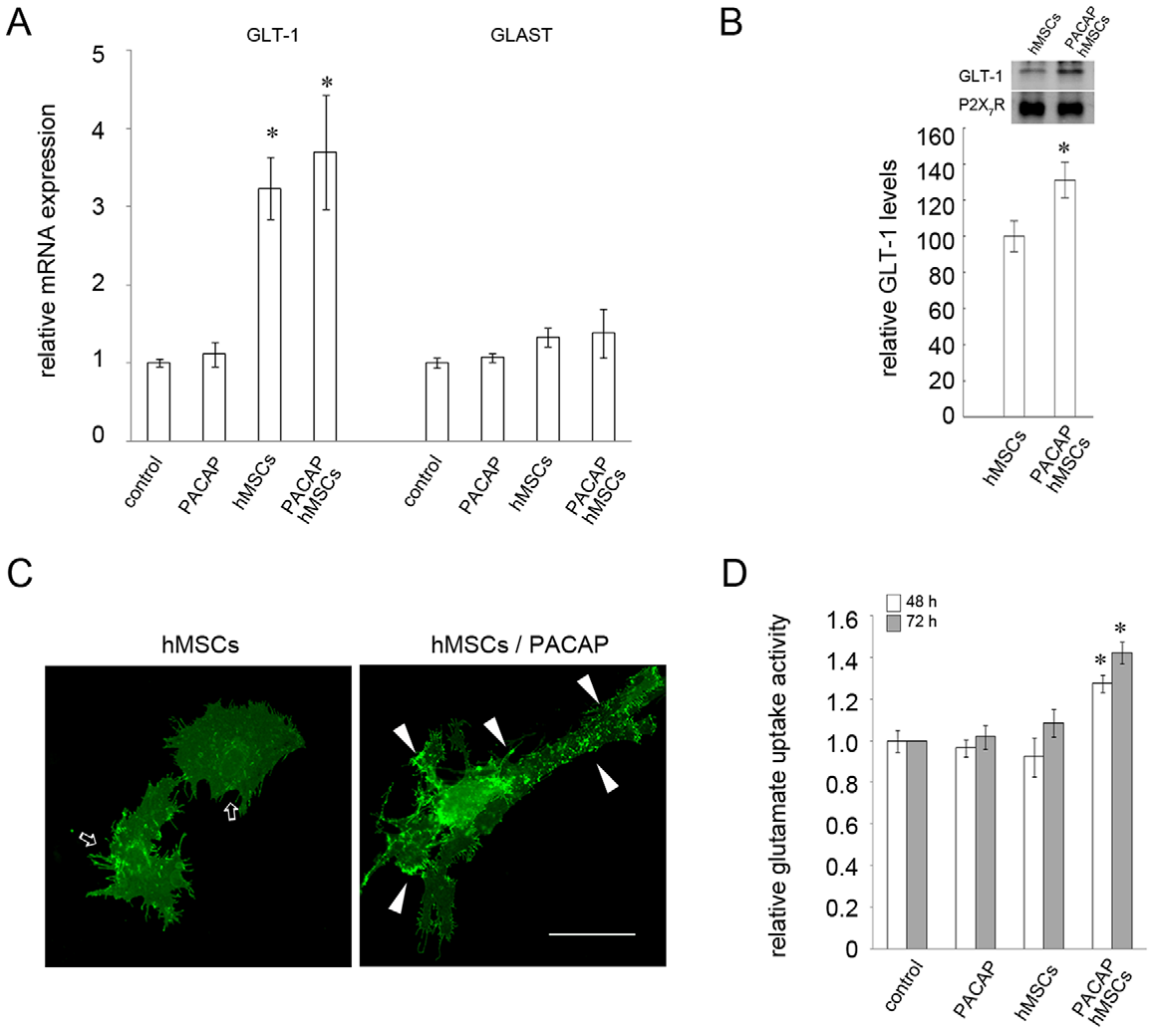
GLT-1 production and glutamate uptake ability of astrocytes promoted by combinatorial treatment with PACAP and hMSCs. (A). Astrocytes were treated with PACAP (20 ng/ml), or indirectly co-cultured with hMSCs in the absence and presence of PACAP (20 ng/ml) for 48 hours. The cultures were subjected to Q-PCR for measurement of the mRNA levels of GLT-1 and GLAST. Data are presented as mean ± SEM and expressed as the ratio of GLT-1 (GLAST) mRNA levels compared to control. *p<0.05 versus control. (B). Astrocytes were indirectly co-cultured with hMSCs in the absence or presence of PACAP for 48 hours. Membrane proteins were then extracted for western blotting using anti-GLT-1 antibody. The same blot was reprobed with anti-P2X_7_R antibody. P2X_7_R levels are presented as a loading control. Relative intensity of GLT-1 levels normalized to P2X_7_R was measured. Data are presented as mean ± SEM and expressed as a percentage of GLT-1 levels in the group with combinatorial treatment compared to that detected in the group co-cultured only with hMSCs. *p<0.05 versus the group only co-cultured with hMSCs. (C). Astrocytes were infected by lentivirus carrying lenti-GLT-1-GFP, and then co-cultured with hMSCs in the absence or presence of PACAP. Strong punctate fluorescence spots (arrowheads) were observed in the processes of astrocytes co-cultured with hMSCs in the presence of PACAP, when compared to that seen in astrocytes co-cultured only with hMSCs (arrows). Scale bar, 50 µm. (D). Astrocytes were treated with PACAP, or co-cultured with hMSCs in the absence or presence of PACAP. After 48 or 72 hours, the cultures were subjected to [^3^H]-L-glutamate uptake analysis as described in Materials and Methods. Data are presented as mean ± SEM and expressed as a ratio of the glutamate uptake in each treated group compared to that detected in the control group. *p<0.05 versus control.

Although reactive astrocytes contribute to impairment of axonal regeneration by the formation of glial scar [31], these cells are essential in the maintenance of CNS function by providing metabolic/trophic/antioxidative support and neurotransmitter homeostasis [32]. Given the fact that the glutamate transporter activity is modulated along alternation in astrocytic morphology [33], we examined whether hMSCs and PACAP mediated astrocytic glutamate uptake activity. At first, Q-PCR was performed for the analysis of the gene expression of the astrocyte-specific glutamate transporters, Na^+^-dependent glutamate-aspartate transporter (GLAST) and GLT-1. Our results revealed that co-culture indirectly with hMSCs in the absence or presence of PACAP profoundly increased the expression of GLT-1 mRNA in astrocytes when compared to that detected in the control culture (Figure 7A). However, no significant change in GLAST mRNA levels was observed after co-culture with hMSCs, application of PACPA, or both.

Interestingly, we found that the cell surface levels of GLT-1 proteins in astrocytes co-cultured with hMSCs were significantly increased by the addition of PACAP (Figure 7B). By GLT-1 gene overexpression approach using lentiviral transduction system, we further confirmed the upregulation of the cell surface localization of GLT-1 in astrocytes by co-cultured with hMSCs in the presence of PACAP. As shown in Figure 7C, diffusible distribution of GLT-1 or few punctate GLT-1 signal (Figure 7C, arrows) was observed in astrocytes co-cultured with hMSCs. However, strong punctate signals were observed in astrocytes transduced with lentiviral vector encoding GLT-1 after astrcoytes were co-cultured with hMSCs in the presence of PACAP (Figure 7C, arrowheads). Moreover, examination of astrocytic glutamate uptake activity indicated that co-culture with hMSCs and PACAP simultaneously improved astrocytes to uptake extracellular glutamate when compared to that observed in the cultures including control, PACAP treatment, or co-culture only with hMSCs (Figure 7D). The results reveal that hMSCs in combination with PACAP increase the membrane levels of GLT-1 which contribute to the improvement of astrocytic glutamate uptake.

## Discussion

In this study, we have demonstrated that the immortalized hMSCs used in this study are able to be differentiated into neuronal-like cells in vitro under the induction of neurogenic factors. However, neurons derived from transplanted hMSCs were not observed in the injured spinal cord. Several studies have indicated that the trans-differentiation of BMSCs failed to be observed in vivo [34], [35], [36]. In addition, the adult mammalian spinal cord is a non-neurogenic site [37], [38]. Thus, it is possible that the injured spinal cord consists of non-permissive environment for neuronal trans-differentiation of transplanted hMSCs.

The trophic support of the hMSCs via secreting growth factors and neurotrophic factors has been well documented [39], [40], [41]. Similarly, our results from antibody arrays have indicated that immortalized hMSCs used in this study were found to produce a high level of angiogenin, EGF, GM-CSF, IGFBP-2/4, and NT-3. Angiogenin, a potent inducer of revascularization, is known to act as a neuroprotective molecule for motoneurons [42]. GM-CSF has been reported to improve functional recovery in rats with SCI via the inhibition of glial scar formation and apoptosis and increased expression of immune cells-produced trophic factors [43], [44], [45]. In addition, NT-3 is considered as a neurotrophic factor of the corticospinal tract and is found to promote corticospinal axon growth and partial functional recovery in rats with mid-thoracic lesions of dorsal columns or dorsal spinal cord hemisection [46]. EGF combined with FGF-2 has also been found to improve functional recovery in rats with mild or moderate clip compression injury at spinal T1 section [47]. Other neuroprotective factors or growth factors, such as BDNF, BMP-4, and PDGF-BB, were also detected in the conditioned medium of hMSCs. Although the expression of inflammatory cytokines, such as IL-1α, IL-1β, and TNFα were detectable, their levels were much lower than those of IL-6. In addition, hMSCs secreted extremely low levels of MCP-2, macrophage inflammatory protein-1 (MIP-1), regulated on activation, normal T-cell expressed and secreted (RANTES), thymus and activation-regulated chemokine (TARC), stromal derived factor-1 (SDF-1), and Eotaxin. The role of IL-6 in spinal cord repair is controversial [48], [49], [50]. Nevertheless, it is thought that inflammation plays the beneficial role in spinal cord repair due to its functions in tissue debris removal and neurotrophic factors secreted by glial cells [51]. Based on the observations from our and other laboratories, implanted hMSCs are expected to provide beneficial niches by secreting those indicated trophic factors for neural cells to survive and grow in the injured spinal cord. Indeed, there were a greater number of remaining neuronal fibers with NF or GAP-43 immunoreactivity in the spinal lesion center of SCI rats that underwent delayed combinatorial treatment with PACAP and hMSCs, when compared to that detected in the vehicle-treated control group.

Peroxiredoxins (Prxs), peroxide-eliminating enzymes ubiquitously expressed in the mammalian cells, consist of six distinct isozymes [52]. The six distinct Prx isozymes are expressed in different brain regions and different cell types. For instance, Prx1 and 6 are exclusively expressed in glial cells, while Prx2, 3, 4 and 5 are profoundly expressed in neurons [53]. We have found that the levels of Prx1, Prx6, and MnSOD in the lesion center of the spinal cord declined along with the longer survival time points, when compared to that detected in sham-operated control (unpublished observations of Wang and Tzeng). The findings from our proteomic analysis and western blotting indicated that increased levels of Prx1, Prx6, and MnSOD detected in the lesion center of the spinal cord from the injured rats treated with PACAP and hMSCs. Perhaps, delayed treatment with PACAP or hMSCs promoted neural cell survival at the lesion center, causing an increase in the levels of the three antioxidation/oxidoreduction-relative proteins at this area. Alternatively, the combinatorial treatment may upregulate the production of the three antioxidation/oxidoreduction-relative proteins in the cells at the lesion site. Thus, the combinatorial treatment with PACAP and hMSCs may provide oxidant defense in the lesion center, which in turn contribute to the spinal cord tissue repair.

No biostatistical difference in the BBB scores was observed between rats receiving transplantation of hMSCs and the sham control group. Yet, the BBB scores of the injured rats with delayed transplantation of PACAP and hMSCs were significantly increased. Moreover, a bundle of elongated neuronal fibers remained in the lesion center of the spinal cord from the injured rats treated with PACAP and hMSCs. A reduction in astrocytic hypertrophy was also observed at the lesion center in the animals that received delayed combinatorial treatment with PACAP and hMSCs. However, the mechanism for the effect of the combinatorial therapy on the improvement of spinal cord tissue repair is not sufficiently understood. Yet, our in vivo observations also demonstrated that there were galectin-3-positive cells with the morphology of Iba-1 positive microglia/macrophages in the injured spinal cord by delayed combinatorial treatment with hMSCs and PACAP. Moreover, we showed that a subpopulation of galectin-3 positive microglia was upregulated when microglia were co-cultured with hMSCs in the presence of PACAP. These galectin-3 expressing cells in the nervous system, including microglia, macrophages, and Schwann cells, are known to be involved in myelin phagocytosis in experimental allergic encephalomyelitis, ischemia, and axonal injury [54], [55], [56], [57]. However, microglia/macrophages without galectin-3 expression do not show effective myelin phagocytosis [58]. Accordingly, our in vivo and in vitro evidence implicates that the combinatorial treatment with hMSCs and PACAP may promote tissue debris clearance by the upregulation of galectin-3-positive phagocytes in the injured spinal cord.

Glutamate is released at remarkably high concentrations by excitatory synaptic transmission in the CNS. However, extracellular glutamate overload results in excessive Ca^2+^ influx into neurons through overactivation of neuronal ionotropic glutamate receptors, causing neuronal excitotoxicity. In general, glutamate homeostasis in the synaptic cleft is maintained by re-uptake of glutamate through neuronal or glial glutamate transporters [59], [60]. However, numerous glutamate-induced CNS neurodegenerative disorders, such as ischemia and traumatic CNS injury, are associated with dysfunction of astrocytic glutamate transporters, GLAST and GLT-1 [61], [62]. In our in vitro study, GLT-1 mRNA levels were upregulated in astrocytes which were co-cultured with hMSCs in the presence or absence of PACAP. However, the glutamate uptake ability of astrocytes and GLT-1 protein levels on the cell surface of astrocytes were significantly increased only by co-culture with hMSCs in the presence of PACAP. The in vitro evidence suggests that the administration of hMSCs with PACAP into the injured spinal cord may increase the cell surface expression of astrocytic GLT-1 proteins to promote clearance of extracellular glutamate and reduce SCI-induced neuroexcitotoxicity.

This study demonstrates the therapeutic potential of delayed combinatorial treatment of PACAP and hMSCs for spinal cord tissue repair in rats with severe SCI. The coordinated effects of PACAP and hMSCs on the increase of phagocytic microglia population and astrocytic glutamate uptaking ability may play the critical role in the promotion of spinal cord tissue repair and hindlimb recovery in rats with severe SCI.

## Materials and Methods

### Cell culture

The plasmid (pTERT-IRES2-EGFP) containing human telomerase (hTERT) and green fluorescence protein (GFP) was constructed by the insertion of a 3.45-kb EcoRI-EcoRI fragment containing the hTERT cDNA into pIRES2-EGFP [63]. The hMSC strain (KP) that was developed by transfection with the type 16 human papilloma virus proteins E6E7 as described previously [23] was further immortalized by nucleoporation with the pTERT-IRES2-EGFP. Single cell clone (3A6) was selected and characterized by examining the gene expression of the E6E7, hTERT and GFP. The cells were then expanded and maintained in DMEM/low glucose (LG) medium supplemented with 10% fetal bovine serum (FBS; Hyclone, Logan, Utah, USA). For the induction of neural differentiation, the immortalized hMSCs were treated with several neurogenic factors in ITS medium that was prepared as described previously [19].

Primary mixed glia isolated from 1 to 2-day-old Sprague-Dawley (SD) rat pup cerebral cortices were prepared as described previously [64]. The cells (10^7^ cells/flask) were then plated onto poly-D-lysine coated T-75 tissue culture flasks. The medium was changed every 2 to 3 days. Eight days later, primary rat microglia were collected using the shake-off method [65]. The remaining cells in the cultures consisted of 92% to 95% primary rat astrocytes (as indicated by GFAP positive staining).

### Spinal cord injury

Severe SCI was induced using the weight-drop device developed at New York University [66], [67]. The animal surgical procedure was performed as reported previously [18], [24], [25]. Briefly, adult female SD rats (250±20 g) were anesthetized with pentobarbital (50 mg/kg). A laminectomy was performed at T9-T10, and the dorsal surface of the spinal cord was compressed by dropping a 10-gm rod from a height of 50 mm. During surgery, the rectal temperature was maintained at 37±0.5°C using a thermostatically-regulated heating pad. Bladder evacuation was applied daily for at least 7 days. Antibiotics (sodium ampicillin, 80 mg/kg) were daily injected into animals for 7–9 days. All surgical interventions and animal care were performed in accordance with the Laboratory Animal Welfare Act, the *Guide for the Care and Use of Laboratory Animals*, and National Cheng Kung University.

### Cell transplantation

At day 7 after contusive injury, the rats received local injection with either PACAP (Calbiochem, Germany), or hMSCs, or both. A 10-µl Exmire microsyringe with a 31-gauge needle was positioned at the midline of the cords 2 mm rostral and caudal (dual injection) to the contusive center. Human MSCs (8–10×10^4^ cells/injection; approximately 2×10^5^ cells/animals) or/and PACAP (1 µg/1 µl/injection; 2 µg/rat) were injected 0.8 mm into the dorsal column of the spinal cord. Less than 10 µl total volume of hMSCs and PACAP was used for one injection. Each injection will be completed within 20 minutes.

### BBB locomotor rating scale

The BBB Locomotor Rating Scale was used to evaluate the hindlimb functional improvement of treated animals with spinal cord contusion [26]. In this study, the behavioral analysis was performed at 31 day post SCI before sacrifice.

### Tissue section preparation

Experimental animals were perfused intracardially with 0.9% cold NaCl (400 ml/rat on average), followed by 4% paraformaldehyde in 0.1 M phosphate buffer (500 ml/rat). Spinal cord tissues were removed, post-fixed in 4% paraformaldehyde overnight, and then cryoprotected in 30% (w/v) sucrose in PBS for one day. The cord (approximately 2 cm in length with the epicenter) was excised, embedded in Tissue Tek OCT (Miles), and then sectioned longitudinally at the thickness of 20 µm for immunofluorescence.

### Immunofluorescence

Spinal tissue sections were rinsed with phosphate-buffered saline (PBS) three times, and then incubated for 30 minutes with 0.1% Triton X-100 in PBS containing 5% horse serum to increase the permeability and reduce nonspecific binding. Primary antibodies were applied to tissue sections at the dilution of 1∶200 overnight at 4°C in a humidified chamber. Sections were rinsed three times with PBS followed by biotinylated secondary antibodies for 1 hour and fluorescein–avidin for 45 minutes at room temperature. For in vitro study, cells were fixed in 4% paraformaldehyde for 10 minutes at room temperature. The cells were incubated with primary antibodies in PBS containing 0.1% Triton X-100 and 5% horse serum overnight at 4°C, followed by incubation with biotinylated secondary antibodies (1∶200; Vector) and FITC-avidin (1∶200; Vector). The antibodies used in this study are listed as follows: anti-synapsin-1 antibodies (1∶200; BD Biosciences, San Diego, CA), α-internexin (1∶200; Chemicon, Temecula, CA), anti-NF-200 kDa (1∶200; NF-200; Sigma, St. Louis, MO), anti-CGRP (1∶200; Chemicon), anti-GAP-43 (1∶200; Chemicon), anti-Iba-1 (1∶200; Wako Pure Chemical, Japan), anti-GFAP (1∶200; Chemicon), anti-galectin-3 (1∶200; ABcam, Cambridge), or anti-HuNu (1∶200; Chemicon). Nuclear staining was carried out using 4′,6-diamidino-2-phenylindole (DAPI) solution. The immunostained cells were visualized under fluorescence microscope (Nikon E800) with a color digital camera or under confocal microscopy.

### Human cytokines antibody array

The hMSCs were seeded onto 35-mm petri-dish (4×10^5^ cells/dish) for 48 hours. The conditioned media were collected for the analysis of trophic factors and cytokines using human cytokine antibody arrays (Ray Biotech, Inc) under the manufacturer's instructions. Briefly, the provided blocking buffer was added to array membranes for 30 minutes. The membranes were then incubated with 1 ml of conditioned media for 2 hours, followed by the addition of biotin-conjugated anti-cytokine antibody for 2 hours. The membranes were incubated with HRP-conjugated streptavidin (1: 100) for another 2 hours, and then were analyzed by chemiluminescence. The membranes were subsequently exposed to Kodak® BioMax™ light film. The intensities of the spots on the array membranes were quantified using NIH ImageJ software (National Institutes of Health, Bethesda, MD).

### Sample preparation for Two-dimensional gel electrophoresis (2-DE)

The spinal segment (4–5 mm) containing the lesion epicenter was homogenized in 0.2 ml of cold lysis buffer consisting of 40 mM Tris, 40 mM sodium acetate, 1% NP-40, 1% Triton X-100, 0.1% SDS, 1 mM phenylmethylsulfonyl fluoride (PMSF; Sigma), and protease inhibitor cocktail in PBS for 30 minutes, followed by sonication. The homogenate was centrifuged at 10,000 g for 30 minutes at 4°C to remove insoluble debris. The proteins were then precipitated by cold acetone with 10% trichloroacetic acid overnight. After centrifugation, the protein pellet was washed with cold acetone followed by air drying, and then resuspended in the rehydration buffer containing 8 M urea, 4% CHAPS, 0.2% Bio-Lyte 3/10 (Bio-Rad, Hercules, CA) and 50 mM dithiothreitol (DTT) (Sigma). Protein concentration was assessed using a Bio-Rad reducing agent and detergent compatible kit (Bio-Rad).

### 2-DE

For the first-dimension isoelectric focusing (IEF), pH 3–10 non-linear range immobilized pH gradients (IPG) strips (11 cm) were rehydrated with 200 µl of solubilized sample (200 µg protein amount) for 12 hours before the sample was separated by IEF at 100 Volt for 30 minutes, 500 Volt for 30 minutes, 1000 Volt for 1 hour, 5000 Volt for 1 hour, and finally 8000 Volt for 3 h. The IPG strips were equilibrated with 2 ml of equilibration buffer consisting of 0.375 M Tris, 6 M urea, 2% SDS, 20% glycerol and 0.02 g/ml DTT at 25°C for 15 minutes followed by equilibration in 0.375 M Tris, 6 M urea, 2% SDS, 20% glycerol and 0.025 g/ml iodoacetamide at 25°C for 15 minutes. The second dimension SDS-PAGE used a 10% separating gel and was then performed without a stacking gel. The equilibrated IPG gel strip was placed on top of the SDS-PAGE gel and was sealed with 0.5% low-melting temperature agarose with 0.01% bromphenol blue as tracking dye. Electrophoresis was carried out at 180 V until the tracking dye reached the bottom of the gel. Silver staining was accessed by immersing the 2-DE gel in the fixation solution containing methanol (50%), acetic acid (12%), and water (38%) for 30 minutes, followed by 50% ethanol for 60 minutes. The gel was sensitized by 0.02% sodium thiosulfate solution and stained in a solution containing 0.2% AgNO_3_ and 0.05% formaldehyde for 20 minutes, and subsequently developed in a solution of 0.05% formaldehyde, 2% Na_2_S_2_O_3_, and 0.0004% Na_2_S_2_O_3_. The staining was stopped at the desired time point by the addition of fixation solution. The spots on the gels were excised, trypsinized, and analyzed by matrix assisted laser desorption ionization time-of-flight mass spectrometer (MALDI-TOF MS) (autoflex III, Bruker Daltonics, Bremen, Germany).

### Western blotting

Spinal tissue blocks (approximately 4–5 mm thickness/block) were taken from the rostral or caudal regions adjacent to epicenter or from the contusive site on the spinal cord. Tissue blocks were homogenized in extraction solution containing 40 mM Tris, 40 mM sodium acetate, and protease inhibitor cocktail (Sigma) using the sonicator. On the other hand, the primary glial cells after treatment were harvested and gently homogenized on ice using PBS containing SDS, 1 mM PMSF, 1 mM ethylenediaminetetraacetic acid (EDTA), 1 mM sodium orthovanadate, and proteinase inhibitor cocktail. Protein concentration was assayed using the Bio-Rad DC kit (Bio-Rad, Hercules, CA). Protein extracts (30 µg/lane) were separated on 10% SDS-PAGE and then transferred to a nitrocellulose filter (Millipore, Billerica, MA). The membrane was probed at 4°C overnight with primary antibodies, and then incubated with horseradish peroxidase (HRP)-conjugated secondary antibodies (Jackson ImmunoResearch Laboratories, West Grove, PA) for 1 hour at room temperature. The detection was carried out by using ECL chemiluminescence (Amersham Pharmacia, Buckinghamshire, United Kingdom). The primary antibodies used in this study are listed as follows: anti-Prx-1 (1∶1000; Abcam), anti-Prx-6 (1∶1000; Abcam), anti-MnSOD (1∶1000; Assay Designs Inc., Ann Arbor, MI), and anti-β-actin (1∶200; Santa Cruz).

### Flow cytometry

The assay was used to measure the expression of galectin-3 in microglia that were seeded on a 35-mm petri-dish at the density of 5×10^5^ per dish and co-cultured with hMSCs (4×10^5^ cells per dish). After treatment, the cultures were incubated in cold PBS containing rabbit anti-galectin3 antibody for 30 minutes at room temperature, followed by FITC-labeled anti-rabbit antibody (1∶100; Vector Labs) for 30 minutes at room temperature. The cells were then subjected to FACScalibur flow cytometer (BD Biosciences). Fluorescent intensity was detected in the FL-1 channel (515–545 nm). Data were analyzed using the WinMDI2.8 (The Scripps Research institute, Flow Cytometry Core Facility, La Jolla, CA). The cytogram of microglia-hMSCs indicated microglia gated in R1, and hMSCs in R2. Values are presented as relative level of galectin-3-positive microglia (R1) in each culture to that detected in the control culture.

### Quantitative real-time polymerase chain reaction (Q-PCR)

The assay was used to measure the mRNA levels of GLT-1 and GLAST in astrocytes, and the method was followed by the procedure described previously [68]. PCR amplification of GLT-1 and GLAST was performed for 10 minutes at 95°C, followed by 50 cycles set for 10 seconds at 95°C, annealing for 10 seconds at 65°C for GLT-1 (or at 60°C for GLAST), and extending for 20 seconds at 72°C. The results were normalized to the housekeeping gene of cyclophilin A (CyPA), and were expressed as a ratio of the percentage of GLT-1 (or GLAST) to the CyPA control treatment.

### Glutamate uptake assay

The experimental procedure was followed as previously described [68]. Astrocyte cultures were incubated at 37°C for 10 minutes in HHBSS assay buffer (in mM: 137 NaCl, 5.4 KCl, 1.26 CaCl_2_, 0.4 MgSO_4_, 0.64 KH_2_PO_4_, 3 NaHCO_3_, 5.5 glucose, and 20 HEPES, pH 7.4) containing 1 µCi/dish of [^3^H]-L-glutamate (Amershan Biosciences) plus 100 µM of unlabeled L-glutamate. The reaction was terminated by aspiration of the assay buffer. The radioactivity of aliquots (350 µl) in 3 ml of liquid scintillation counting solution was measured by a Liquid Scintillation Counter. In parallel, 10-µl aliquots were taken to measure the protein content using a Bio-Rad DC protein assay kit. The radioactivity after normalization to the protein content in the cultures was referred as glutamate uptake activity. The relative uptake activity was represented as the percentage of the uptake activity of the treated culture over the control group.

### Preparation of glutamate transporter-1 expressing vector and lentiviral vector transduction

According to cDNA sequences of rat GLT-1 (NM-017215) in Genbank, the primers (sense: 5′- GCCATGGCATCAACCGAGGGT-3′; GLT-1 anti-sense: 5′-TTTTTCACGTTTCCAAGGTTCT-3′) of full length GLT-1 was designed. Total RNA was isolated from adult SD rat cortical tissues, and then subjected to RT-PCR using GLT-1 primers with 35-cycle reaction at 94°C for 30 seconds, 62°C for 30 seconds, and 72°C for 1 minute 30 seconds. GLT-1 cDNA product (a 1721-bp fragment; +109 to +1830) was cloned into TA cloning vector (TOPO II, Invitrogen). The orientation of the cDNA inserts was verified by autosequencing. The cDNA inserts were cut out using BamHI and XhoI and subcloned into pLV-CAG-GLT-1-GFP lentiviral vector (lenti-GLT-1-GFP). The lentiviral vectors expressing GLT-GFP were constructed and the lentivirus particles were prepared by Biosettia, Inc (San Diego, CA, USA). Astrocytes (4×10^5^ cells/well) were replated onto 12-mm coverslips placed in a 24-well plate for 2–3 days, and then were transduced by the appropriate amount (150 µl virus particles) of lenti-GLT-1-GFP transduction particles with DMEM/F-12 medium containing 10% calf serum (Hyclone). Twenty-four hours after the transduction, the culture medium was replaced with fresh DMFM/F-12 medium containing 10% CS, and the cell cultures were incubated for another 48 hours. Infected astrocytes were then co-cultured indirectly with hMSCs. The hMSCs (5×10^4^ cells per well) were seeded onto the Transwells with Millicell-CM filters (12 mm in diameter; pore size 0.4 µm) for 24 hours before co-culture was performed. After 72 hours, the cultures were washed with PBS for three times, followed by 4% paraformaldehyde in PBS for 15 minutes at room temperature.

### Analysis of cell surface levels of glutamate transporter-1

Primary astrocytes were cultured at the density of 4×10^5^ cells per 35 mm petri dish and indirectly co-incubated with hMSCs (5×10^5^ cells per filter) that were plated onto the Transwells with Millicell-CM filters (30 mm in diameter) with 0.4-µm pores for 24 hours. After treatment by PACAP for 48 hours, membrane proteins were extracted using ProteoJET™ membrane protein extraction kit, according to manufacturer's instructions (Fermentas, EU). Protein concentration was determined using a Bio-Rad DC kit (Hercules, CA). Fifty micrograms of membrane proteins were loaded onto 7.5% SDS-PAGE and transferred to a nitrocellulose membrane. The protein was identified by incubating the membrane with anti-GLT-1 antibody (1∶1000; Millipore) or anti- purinergic P2X_7_R antibody (1∶1000; Alomone Labs, Israel) overnight at 4°C, followed by HRP-conjugated secondary antibody (1∶2000) and ECL solution.

### Statistical Analysis

The results are presented as mean ± SEM. The two tailed Mann-Whitney test and repeated measures analysis of variance were performed to evaluate the statistical significance of the results (*p* value <0.05).

## Supporting Information

Table S1 **A list of antioxidation- and signal transduction-related protein spots identified by in-gel digestion and MALDI-TOF MS analysis.** Induced changes in the expression of selected proteins by combinatorial treatment with PACAP and hMSCs are indicated by up-arrows (increase) and down-arrows (decrease) when compared to that observed in vehicle-treated group.
(DOC)

Figure S1 **Separation of proteins from the lesion center of vehicle-treated injured spinal cord by 2-DE.** The spinal cord tissues were dissected from the lesion center at 1 week after transplantation, and digested in the detergent containing lysis buffer consisting of 40 mM Tris, 40 mM sodium acetate, 1% NP-40, 1% Triton X-100, 0.1% SDS, 1 mM PMSF, and protease inhibitor cocktail in PBS for 30 min, followed by sonication. Total proteins (200 µg) of the soluble fractions were separated by 2-DE and subjected to MALDI-TOF analysis. Total 142 proteins were identified through MALDI-TOF mass spectrometry and subsequent database searching.
(DOC)
