# Supplementary material for: Effects of Combinatorial Treatment with Pituitary Adenylate Cyclase Activating Peptide and Human Mesenchymal Stem Cells on Spinal Cord Tissue Repair
Source: PLoS One. 2010 Dec 20;5(12):e15299. doi: 10.1371/journal.pone.0015299 (PMC3004866; doi:10.1371/journal.pone.0015299)
Supplement: Table S1 — A list of antioxidation- and signal transduction-related protein spots identified by in-gel digestion and MALDI-TOF MS analysis. Induced changes in the expression of selected proteins by combinatorial treatment with PACAP and hMSCs are indicated by up-arrows (increase) and down-arrows (decrease) when compared to that observed in vehicle-treated group. (DOC) [file pone.0015299.s001.doc]

**Table S1**

**A list of antioxidation- and signal transduction-related protein spots identified by in-gel digestion and MALDI-TOF MS analysis.** Induced changes in the expression of selected proteins by combinatorial treatment with PACAP and hMSCs are indicated by up-arrows (increase) and down-arrows (decrease) when compared to that observed in vehicle-treated group.

| **Spot  No.** | **Accession No.** | **Protein name** | **Function** | **Expression** | **(M**_r_) **/ pI** | **Match  score** | **Match Peptide** | **Sequence  Converage** |
| --- | --- | --- | --- | --- | --- | --- | --- | --- |
| 63 | P04762 | Catalase | Antioxidation/ oxidoreduction | ↓ | 59719/7.07 | 184 | 16 | 41% |
| 125 | Q68FU3 | Electron transfer flavoprotein subunit beta | Antioxidation/ oxidoreduction | ↓ | 27670/7.6 | 74 | 6 | 34% |
| 39 | P11884 | Aldehyde dehydrogenase, mitochondrial | Antioxidation/ oxidoreduction | ↓ | 56453/6.63 | 95 | 12 | 26% |
| 126 | Q923D2 | Flavin reductase | Antioxidation/ oxidoreduction | ↓ | 22183/6.49 | 79 | 8 | 50% |
| 45 | O08651 | D-3-phosphoglycerate dehydrogenase | Antioxidation/ oxidoreduction | ↑ | 56457/6.28 | 110 | 10 | 26% |
| 94 | P35704 | Peroxiredoxin-2 | Antioxidation/ oxidoreduction | ↑ | 21770/5.34 | 82 | 6 | 23% |
| 106 | O35244 | Peroxiredoxin-6 | Antioxidation/ oxidoreduction | ↑ | 24803/5.64 | 90 | 6 | 20% |
| 26, 27, 28 | P11598 | Protein disulfide-isomerase A3 | Antioxidation/ oxidoreduction | ↑ | 56588/5.88 | 182 | 17 | 38% |
| 129, 131 | Q63716 | Peroxiredoxin-1 | Antioxidation/ oxidoreduction | ↑ | 22095/8.27 | 92 | 9 | 37% |
| 130 | P07895 | Superoxide dismutase [Mn], mitochondrial | Antioxidation/ oxidoreduction | ↑ | 24659/8.96 | 79 | 7 | 43% |
| 95 | P19132 | Ferritin heavy chain | Antioxidation/ oxidoreduction | ↑ | 21113/5.85 | 72 | 6 | 41% |
| 100 | P07943 | Aldose reductase | Antioxidation/ oxidoreduction | ↑ | 35774/6.26 | 62 | 7 | 32% |
| 110 | P51635 | Alcohol dehydrogenase [NADP+] | Antioxidation/ oxidoreduction | ↑ | 36483/6.84 | 108 | 10 | 39% |
| 40, 41 | P04764 | Alpha-enolase | Antioxidation/ oxidoreduction | ↑ | 47098/6.16 | 179 | 18 | 52% |
| 19 | P04785 | Protein disulfide-isomerase | Antioxidation/ oxidoreduction | ↑ | 56916/4.82 | 98 | 11 | 25% |
| 105 | P60901 | Proteasome subunit alpha type-6 | Signal transduction | ↓ | 27382/6.34 | 66 | 7 | 36% |
| 101 | P14669 | Annexin A3 | Signal transduction | ↓ | 36341/5.96 | 98 | 8 | 26% |
| 118 | P63245 | Guanine nucleotide-binding protein subunit beta-2-like 1 | Signal transduction | ↓ | 35055/7.6 | 75 | 6 | 24% |
| 10 | P50398 | Rab GDP dissociation inhibitor alpha | Signal transduction | ↑ | 50504/5.0 | 77 | 10 | 33% |
| 121 | P08699 | Galectin-3 | Signal transduction | ↑ | 27184/8.59 | 73 | 7 | 21% |
| 90 | Q5XI73 | Rho GDP-dissociation inhibitor 1 | Signal transduction | ↑ | 23393/5.12 | 72 | 7 | 36% |
| 92, 93 | P31044 | Phosphatidylethanolamine-binding protein 1 | Signal transduction | ↑ | 20788/5.48 | 66 | 7 | 51% |
