# Supplementary material for: Effects of Combinatorial Treatment with Pituitary Adenylate Cyclase Activating Peptide and Human Mesenchymal Stem Cells on Spinal Cord Tissue Repair
Source: PLoS One. 2010 Dec 20;5(12):e15299. doi: 10.1371/journal.pone.0015299 (PMC3004866; doi:10.1371/journal.pone.0015299)
Supplement: Figure S1 — Separation of proteins from the lesion center of vehicle-treated injured spinal cord by 2-DE. The spinal cord tissues were dissected from the lesion center at 1 week after transplantation, and digested in the detergent containing lysis buffer consisting of 40 mM Tris, 40 mM sodium acetate, 1% NP-40, 1% Triton X-100, 0.1% SDS, 1 mM PMSF, and protease inhibitor cocktail in PBS for 30 min, followed by sonication. Total proteins (200 µg) of the soluble fractions were separated by 2-DE and subjected to MALDI-TOF analysis. Total 142 proteins were identified through MALDI-TOF mass spectrometry and subsequent database searching. (DOC) [file pone.0015299.s002.doc]

**Figure S1**

**Separation of proteins from the lesion center of vehicle-treated injured spinal cord by 2-DE.**


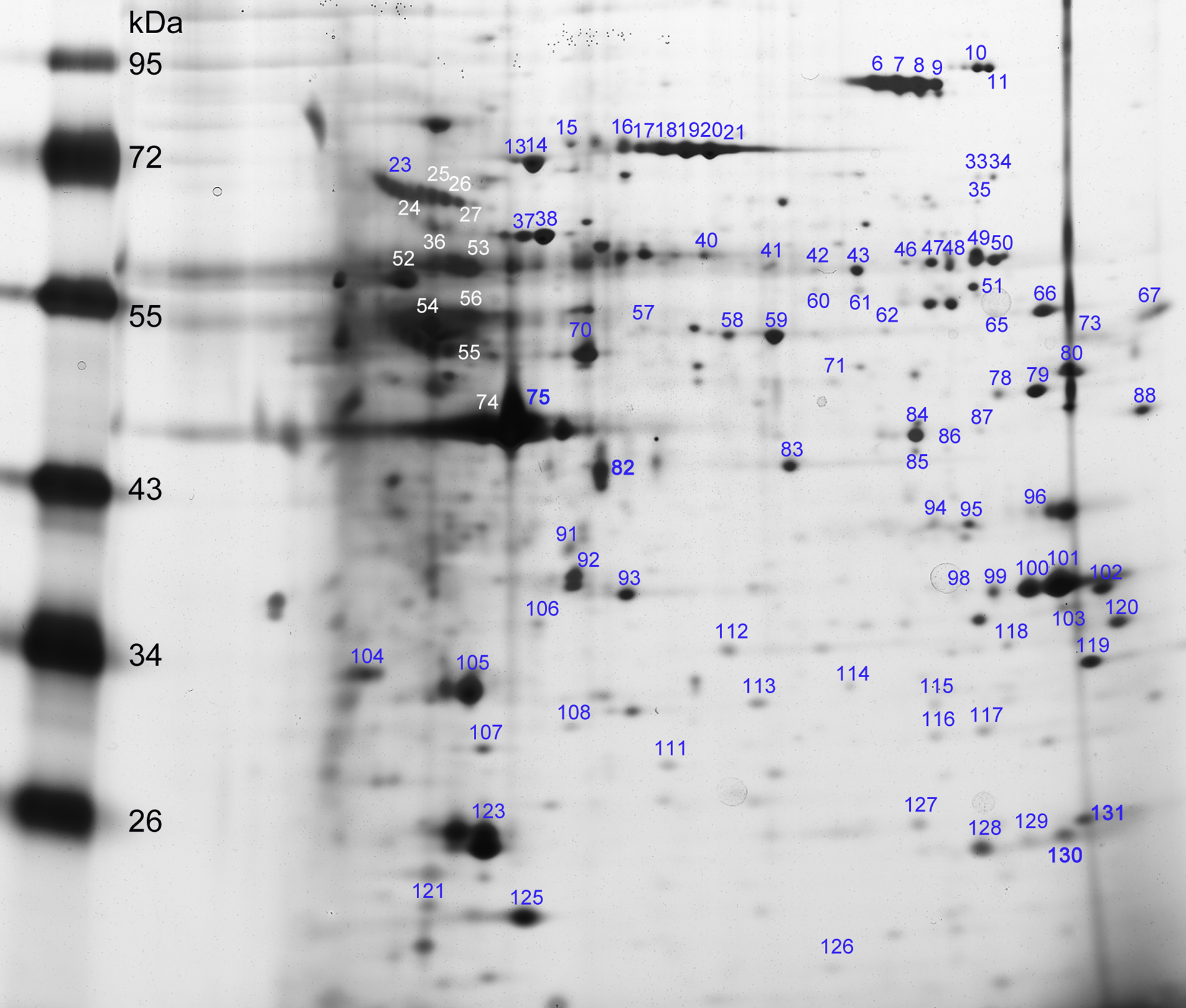


The spinal cord tissues were dissected from the lesion center at 1 week after transplantation, and digested in the detergent containing lysis buffer consisting of 40 mM Tris ,40 mM sodium acetate, 1% NP-40, 1% Triton X-100, 0.1% SDS, 1 mM PMSF, and protease inhibitor cocktail in PBS for 30 min, followed by sonication. Total proteins (200 μg) of the soluble fractions were separated by 2-DE and subjected to MALDI-TOF analysis. Total 142 proteins were identified through MALDI-TOF mass spectrometry and subsequent database searching
